# Supplementary material for: The cholesterol biosynthesis pathway regulates IL-10 expression in human Th1 cells
Source: Nat Commun. 2019 Jan 30;10:498. doi: 10.1038/s41467-019-08332-9 (PMC6353904; doi:10.1038/s41467-019-08332-9)
Supplement: Supplementary file 1 — Supplementary Information [file 41467_2019_8332_MOESM1_ESM.pdf]

The cholesterol biosynthesis pathway regulates IL-10 expression in human Th1 cells.

Perucha E et al.

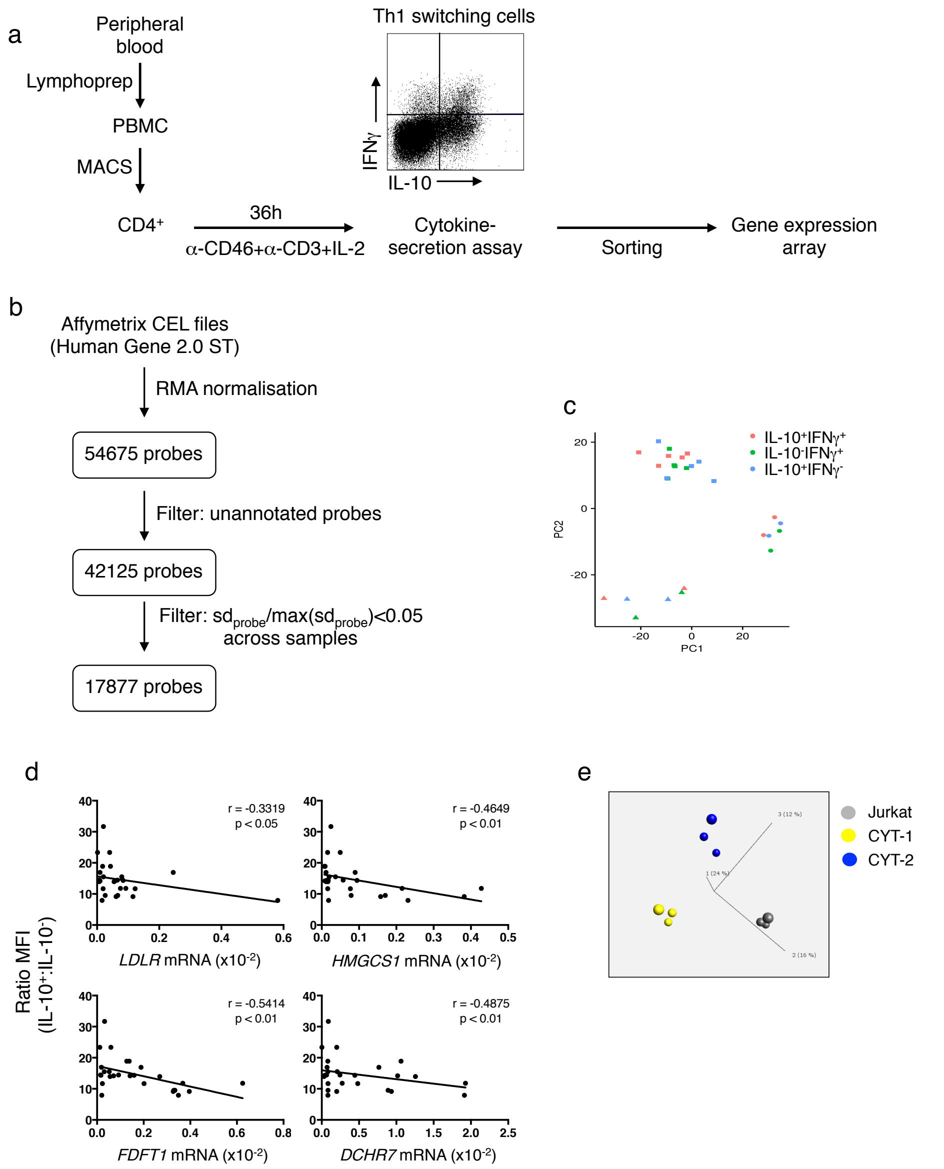

Supplementary Figure 1. Metabolic regulation of Th1 switching in primary human $CD4^+$ T cells. (**a**) Schematic representation of the experimental set-up leading to Th1 switching cells and gene expression array experiments. (**b**) Schematic representation of the micro-array analysis workflow. (**c**) Clustering analysis of gene expression data on $IFN\gamma^+IL\text{-}10^-$ (green), $IFN\gamma^+IL\text{-}10^+$ (red) and $IFN\gamma^-IL\text{-}10^+$ (blue) populations. (**d**) Relation between mRNA levels of cholesterol biosynthesis pathway selected targets in the $IFN\gamma^-IL\text{-}10^-$ and $IFN\gamma^+IL\text{-}10^-$ populations and the MFI ratio for IL-10 ($IFN\gamma^+IL\text{-}10^+$:$IFN\gamma^-IL\text{-}10^-$) (n=14), showing a linear regression for each gene. R and p values for one-tailed Spearman test for each target are shown. (**e**) Principal component analysis of gene expression array derived from control Jurkat T cells *versus* Jurkat-CYT-1 *versus* Jurkat-CYT-2 expressing cells.

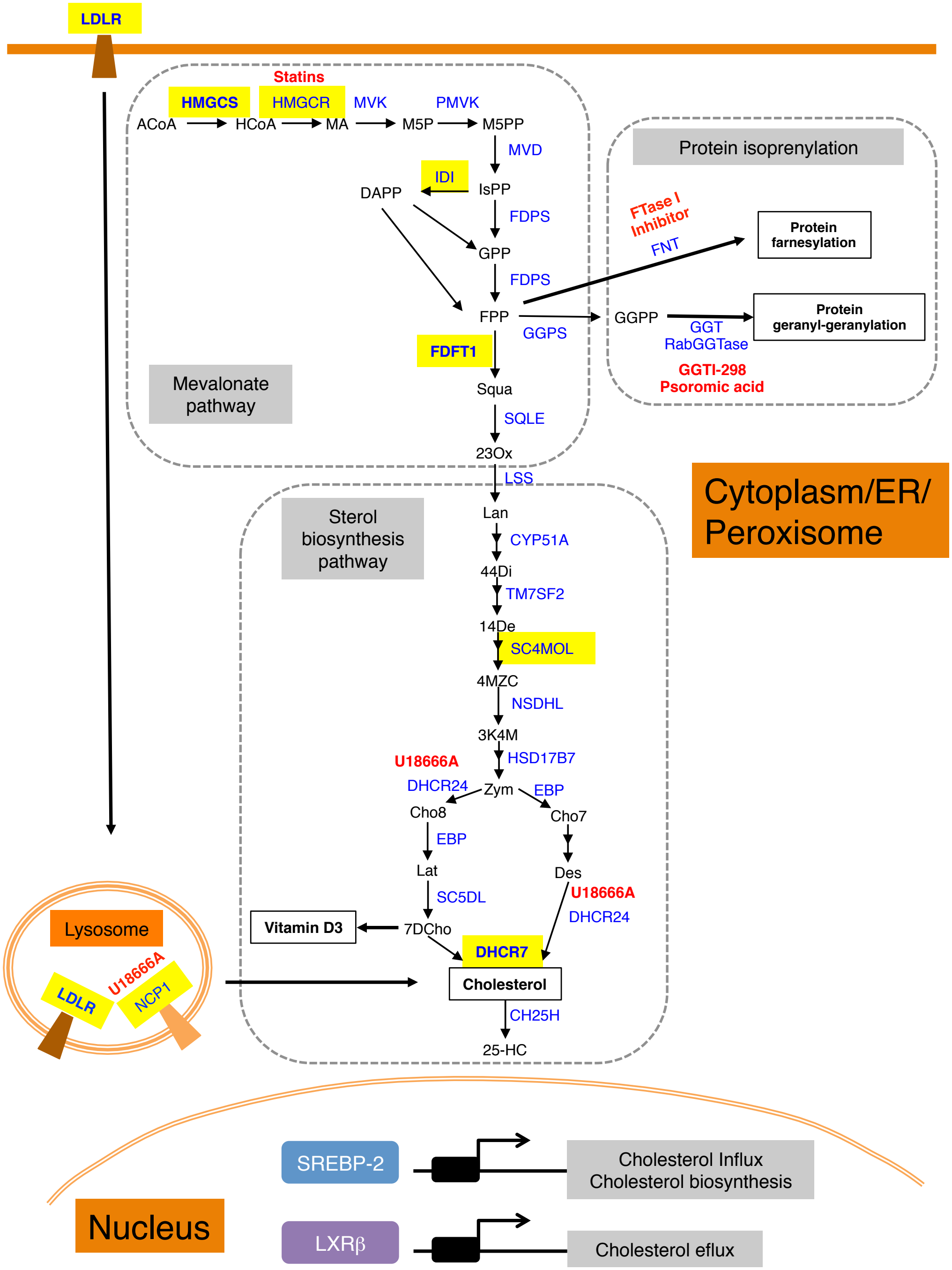

Supplementary Figure 2. Schematic representation of the cholesterol biosynthesis pathway and its regulation. Metabolites of the pathway are shown in black, enzymes of the pathway are shown in blue and inhibitors are shown in red. The pathway is regulated by two master transcription factors: Liver X receptor β (LXRβ) negatively regulates the pathway by promoting cholesterol efflux while Sterol Regulatory Element-Binding Protein-2 (SREPB-2) positively regulates the pathway by promoting the transcription of the enzymes implicated in the pathway as well as cholesterol import via the LDLr. Probes significantly associated with IL-10 levels (Fig. 1b) are highlighted in yellow. Legend with full names is provided in Supplementary Table 1.

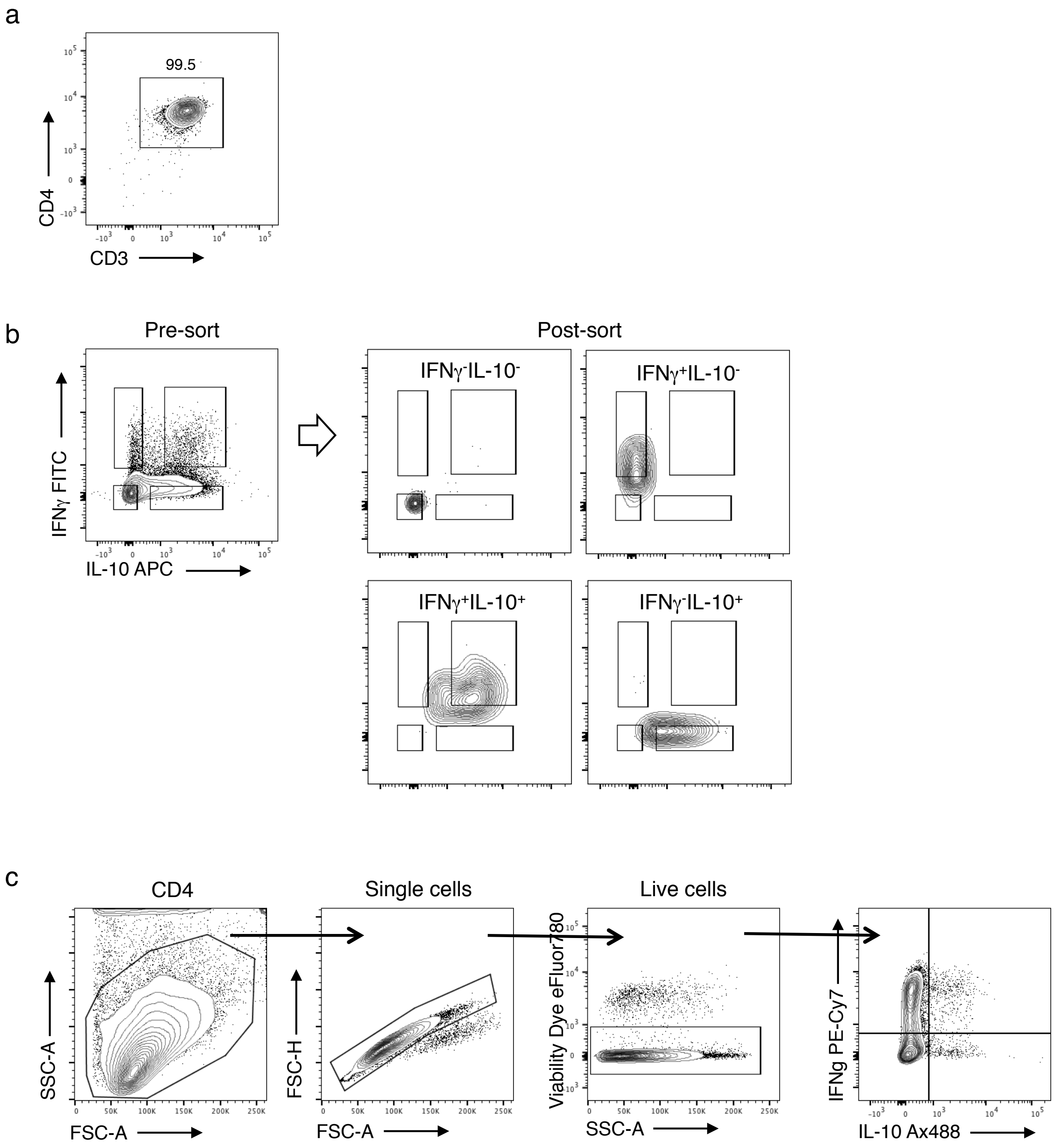

Supplementary Figure 3. Gating strategies. Purified human CD4$^+$ T cells stimulated *in vitro* with plate bound α-CD3 (2 μg ml^{-1}) + α-CD46 (5 μg ml^{-1}) + rhIL-2 (50 U ml^{-1}) were cultured for 36 hours. (**a**) Representative CD4$^+$ purity gating. (**b**) Gating strategy and post-sort analysis for cytokine secretion assay presented in Fig. 1 and Supplementary Fig. 1. (**c**) Gating strategy for intracellular cytokine staining analysis presented in Fig. 2, 3, 4 and Supplementary Fig. 4, 6.

.

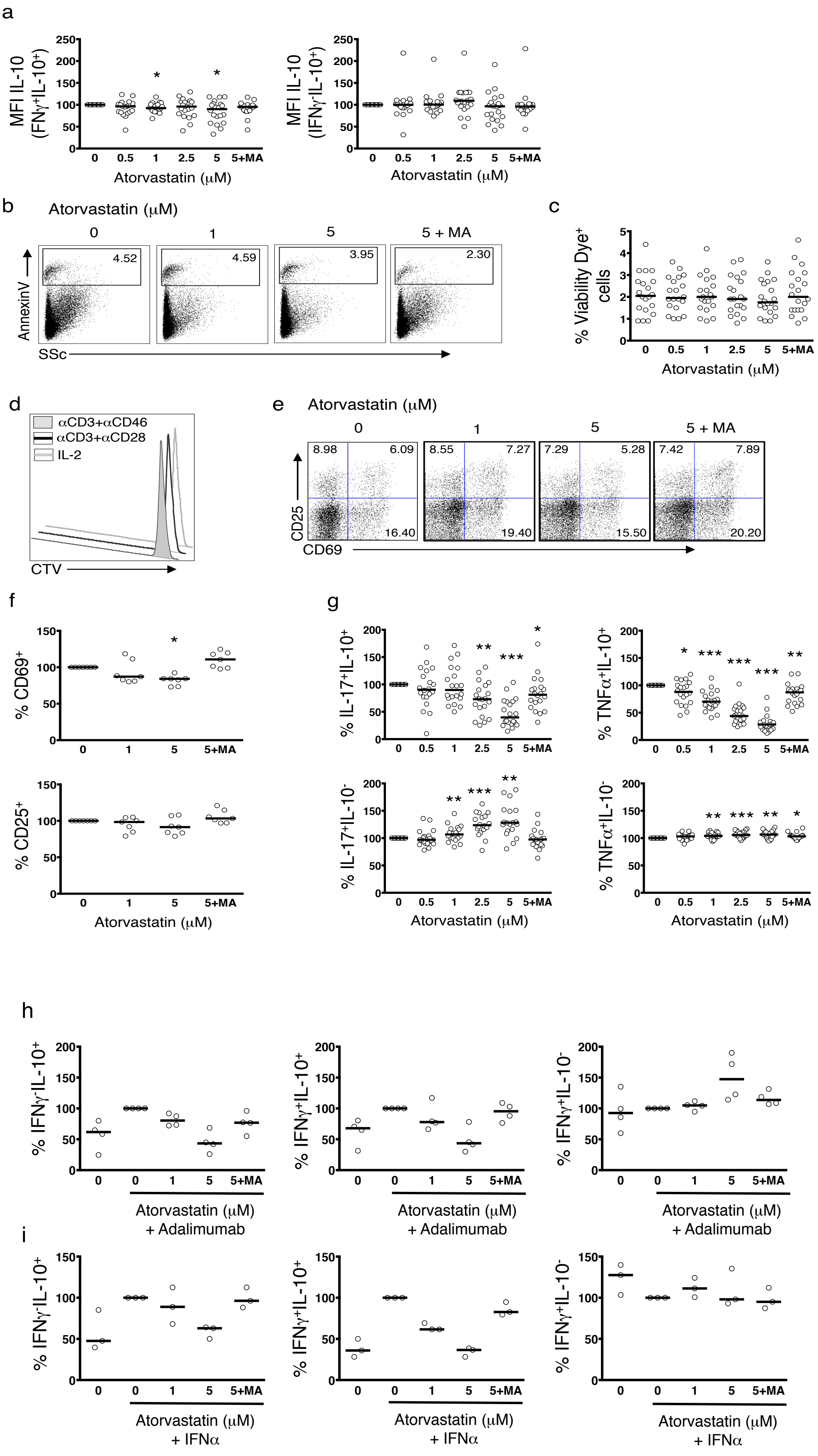
a
MFI IL-10 (IFNγ+IL-10+)
MFI IL-10 (IFNγ-IL-10+)
Atorvastatin (μM)
0 0.5 1 2.5 5 5+MA
b
Atorvastatin (μM)
0 1 5 5 + MA
4.52 4.59 3.95 2.30
AnnexinV
SSc
c
% Viability Dye+ cells
d
αCD3+αCD46
αCD3+αCD28
IL-2
CTV
e
8.98 6.09 16.40
8.55 7.27 19.40
7.29 5.28 15.50
7.42 7.89 20.20
CD25
CD69
f
% CD69+
% CD25+
g
% IL-17+IL-10+
% TNFα+IL-10+
% IL-17+IL-10-
% TNFα+IL-10-
h
% IFNγ-IL-10+
% IFNγ+IL-10+
% IFNγ+IL-10-
Atorvastatin (μM) + Adalimumab
i
Atorvastatin (μM) + IFNα

Supplementary Figure 4. Th1 switching to IL-10 is blocked when the mevalonate pathway is inhibited. Purified human CD4$^+$ T cells stimulated *in vitro* with plate bound α-CD3 (2 μg ml^{-1}) + α-CD46 (5 μg ml^{-1}) + rhIL-2 (50 U ml^{-1}) were cultured for 36 hours in the presence of atorvastatin and 250 μM mevalonic acid (MA) as indicated, unless stated otherwise. (**a**) Normalised mean fluorescence intensity (MFI) for IL-10 in IFNγ$^+$IL-10$^+$ (left) and IFNγ$^-$L-10$^+$ (right) cells (n=20). (**b**) Representative dot-plots for annexin staining on treated cells (data are representative of 5 independent donors). (**c**) Percentage of fixable viability dye positive cells (n=20). (**d**) Representative histogram of Cell Trace Violet (CTV) staining of CD4$^+$ T cells stimulated in vitro with IL-2, α-CD3 + α-CD46 or beads coated with α-CD3 + α-CD28 for 36 hours (data are representative of 3 independent donors). (**e**) Representative dot-plot of CD69$^+$ and CD25$^+$ surface staining. (**f**) Normalised frequency of CD69$^+$ (left) and CD25$^+$ (right) cells (n=7). (**g**) Normalised frequency of IL-17$^+$IL-10$^+$ (top left), IL-17$^+$IL-10$^-$ (bottom left), TNFα$^+$IL-10$^+$ (right left), TNFα$^+$IL-10$^-$ (bottom right) producing cells (n=20). (**h**) Normalised frequency of IL-10$^+$IFNγ$^-$ (left), IFNγ$^+$L-10$^+$ (centre) and IFNγ$^+$IL-10$^-$ (right) CD4$^+$ T cells co-cultured with monocytes and α-CD3 (100 ng ml^{-1}) in the absence or presence of Adalimumab (1 μg ml^{-1}) and treated with atorvastatin and MA (n=4). (**i**) Normalised frequency of IL-10$^+$IFNγ$^-$ (left), IFNγ$^+$L-10$^+$ (centre) and IFNγ$^+$IL-10$^-$ (right) CD4$^+$ T cells activated for 3 days with plate bound α-CD3 (0.5 μg ml^{-1}) + α-CD28 (1 μg ml^{-1}) + IFNα (600 U ml^{-1}) and treated with atorvastatin and MA (n=3). Graphs show independent donors (dots) normalised to 0 dose of atorvastatin; bars represent median values. *<0.05, **<0.01 and ***<0.001 denote a significant difference compared to untreated cells by repeated measures one-way ANOVA test with *post hoc* Dunnett's correction (a, c, g) or Friedman test with *post hoc* Dunn's correction (f).

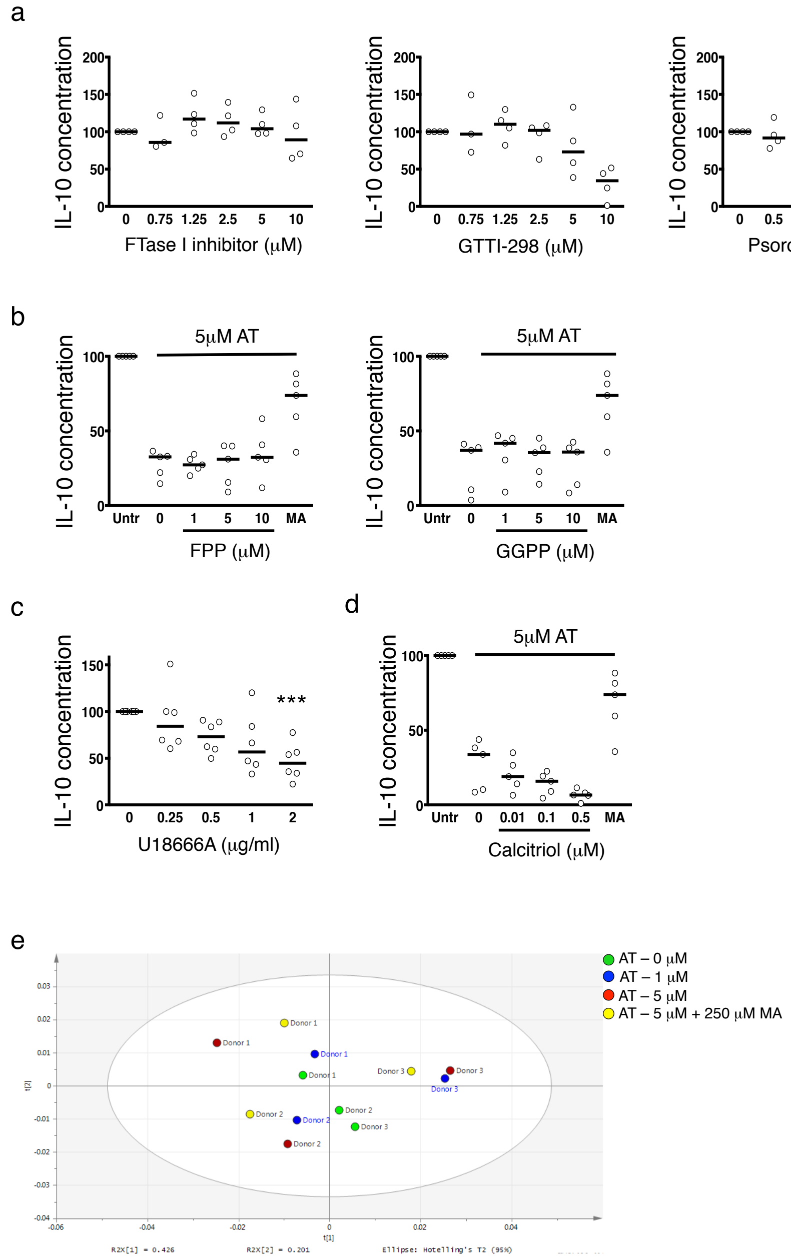

Supplementary Figure 5. IL-10 regulation in Th1 switching cells is dependent on fit CBP metabolism, but not on isoprenylation, vitamin D3 or cellular cholesterol content. Purified human CD4$^+$ T cells stimulated *in vitro* with plate bound α-CD3 (2 μg ml^{-1}) + α-CD46 (5 μg ml^{-1}) + rhIL-2 (50 U ml^{-1}) were cultured for 36 hours in the presence of selected metabolites and inhibitors as indicated. (**a**) Normalised concentrations of secreted IL-10 by cells cultured in the presence of inhibitors for farnesyl-PP transferase (FTase I inhibitor) (left), geranylgenaryl-PP synthase (GGTI-298) (centre) and Rab geranylgeranyltransferase (psoromic acid) (right) (n=4). (**b**) Normalised concentrations of secreted IL-10 by cells cultured with 5 μM atorvastatin (AT) and increasing concentrations of farnesylpyrophosphate (FPP) (left) and geranylgeranylphyrophosphate (GGPP) (right) and mevalonic acid (MA) as control (n=5). (**c**) Normalised concentrations of secreted IL-10 by cells cultured in the

presence of increasing concentrations of U1866A (n=6). (**d**) Normalised concentrations of secreted IL-10 by cells cultured with 5 μM atorvastatin (AT) and increasing concentrations of calcitriol and mevalonic acid (MA) as control (n=5). (**e**) Principal component analysis of lipidomics analysis of cells treated with atorvastatin (AT) and mevalonic acid (MA) (n=3). Graphs show independent donors (dots) normalised to untreated cells; bars represent median values. *P* values: ***<0.001 denote a significant difference compared to untreated cells by Friedman test with *post hoc* Dunn's correction for multiple comparisons.

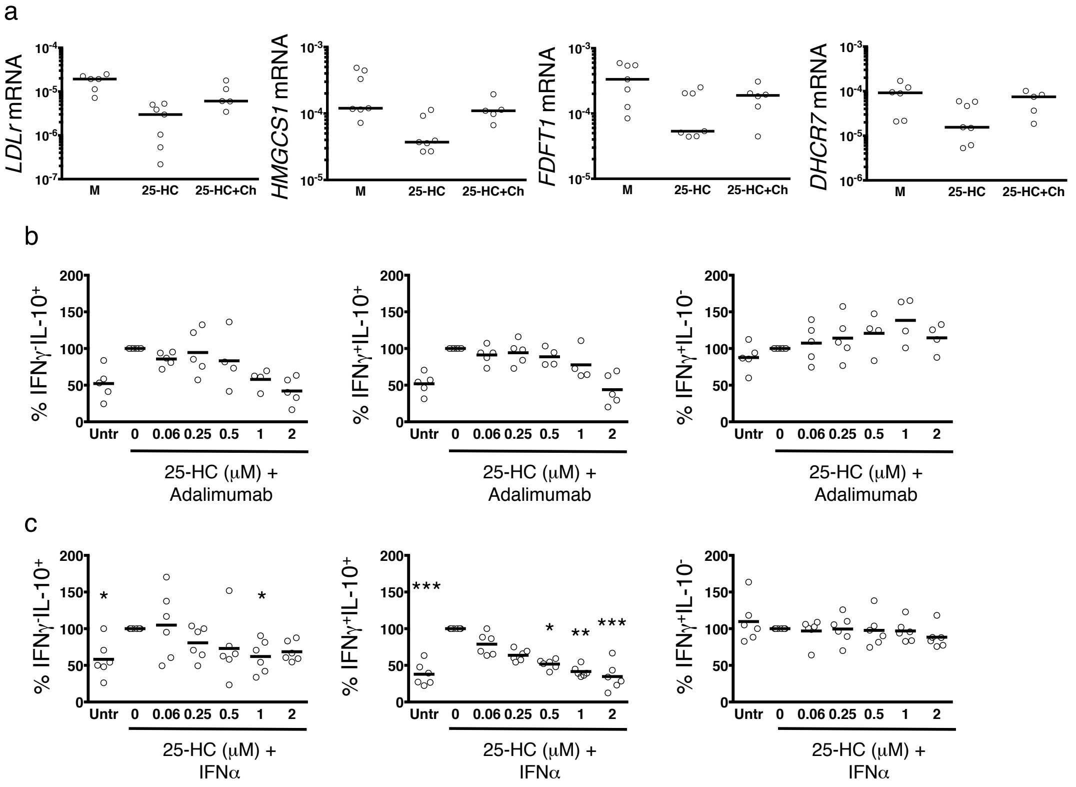

Supplementary Figure 6. IL-10 expression in Th1 switching cells is dependent on intact cholesterol pathway fitness. Purified human CD4$^+$ T cells stimulated *in vitro* with plate bound α-CD3 (2 μg ml^{-1}) + α-CD46 (5 μg ml^{-1}) + rhIL-2 (50 U ml^{-1}) were cultured for 36 hours in the presence of 25-hydroxycholesterol (25-HC) and different media conditions as indicated. (**a**) Normalised *LDLr*, *HMGCS1*, *FDFT1* and *DHCR7* mRNA levels of cells cultured in fully supplemented media (M), 25-HC (2 μM) with or without cholesterol 500x (Ch) (n=5-7). (**b**) Normalised frequency of IL-10$^+$IFNγ$^-$ (left), IFNγ$^+$L-10$^+$ (centre) and IFNγ$^+$IL-10$^-$ (right) CD4$^+$ T cells co-cultured with monocytes and α-CD3 (100 ng ml^{-1}) in the absence or presence of Adalimumab (1 μg ml^{-1}) and treated with 25-HC (n=5). (**c**) Normalised frequency of IL-10$^+$IFNγ$^-$ (left), IFNγ$^+$L-10$^+$ (centre) and IFNγ$^+$IL-10$^-$ (right) CD4$^+$ T cells activated for 3 days with plate bound α-CD3 (0.5 μg ml^{-1}) + α-CD28 (1 μg ml^{-1}) + IFNα (600 U ml^{-1}) and treated with 25-HC (n=6).

Supplementary table 1: Supplementary Figure 2 legend.

Metabolites

ACoA	Acetyl-CoA
HCoA	3-hydroxy-3-methylglutaryl-CoA
MA	Mevalonic Acid
M5P	Mevalonate-5-phosphate
M5PP	Mevalonate-5-pyrophosphate
IsPP	Isopentenyl-5-pyrophosphate
DAPP	Dimethylallyl-PP
GPP	Geranyl-PP
FPP	Farnesyl-PP
GGPP	Geranylgeranyl-PP
Squa	Squalene
23Ox	2,3-oxidosqualene
Lan	Lanosterol
44Di	4,4'-dimethyl-cholesta-8,14,24-trieneol
14De	14-demethyl-lanosterol
4MZC	4-methylzymosterol-carboxylate
3K4M	3-keto-4-methyl-zymosterol
Zym	Zymosterol
Cho7	Cholesta-7,24-dien-3beta-ol
Des	Desmosterol
Cho8	Cholesta-8,en-3beta-ol
Lat	Lathosterol
7DCho	7-dehydrocholesterol
25HC	25-hydroxycholesterol

Enzymes/proteins

HMGCS	3-hydroxy-3-methylglutaryl-CoA synthase
HMGCR	3-hydroxy-3-methylglutaryl-CoA reductase
MVK	Mevalonate Kinase
PMVK	Phosphomevalonate kinase
MVD	Mevalonate-5-pyrophosphate decarboxilase
FDPS	Farnesyl-PP synthase
IDI	Isopentenyl-PP isomerase
FNT	Farnesyltransferase
GGPS	Geranylgeranyl-PP synthase
GGT	Geranylgeranyl transferase
RabGGTase	Rab geranylgeranyl transferase
FDFT1	Farnesyl-PP farnesyltransferase 1 (squalene synthase)
SQLE	Squalene epoxydase
LSS	Lanosterol synthase
CYP51A	Lanosterol 14 alpha-demethylase
TM7SF2	Delta(14)-sterol reductase
SC4MOL (MSMO1)	Methylsterol Monooxygenase 1

NSDHL	Sterol-4-alpha-carboxylate 3-dehydrogenase
HSD17B7	3-ketosteroid reductase
DHCR24	24-dehydrocholesterol reductase
EBP	Sterol isomerase
SCD5L	Lathosterol oxidase
DHCR7	7-dehydrocholesterol reductase
CH25H	25-hydroxycholesterol hydroxylase
NPC1	Niemann-Pick disease, type C1
LDLR	Low density lipoprotein receptor
