# Supplementary material for: The cholesterol biosynthesis pathway regulates IL-10 expression in human Th1 cells
Source: Nat Commun. 2019 Jan 30;10:498. doi: 10.1038/s41467-019-08332-9 (PMC6353904; doi:10.1038/s41467-019-08332-9)
Supplement: Supplementary file 4 — Description of Additional Supplementary Files [file 41467_2019_8332_MOESM4_ESM.docx]

Description of Additional Supplementary Files

**Supplementary Data 1**: Genes in IL-10 negative populations correlated with IL-10 levels in double positive cells.

**Supplementary Data 2**: Lipidomic analysis of CD4+ T cells treated with atorvastatin ± mevalonic acid.
